# A deep learning approach for electric motor fault diagnosis based on modified InceptionV3

**DOI:** 10.1038/s41598-024-63086-9

**Published:** 2024-05-29

**Authors:** Lifu Xu, Soo Siang Teoh, Haidi Ibrahim

**Affiliations:** School of Electrical and Electronic Engineering, USM Engineering Campus, Universiti Sains Malaysia, 14300 Nibong Tebal, Malaysia

**Keywords:** Electrical and electronic engineering, Computer science

## Abstract

Electric motors are essential equipment widely employed in various sectors. However, factors such as prolonged operation, environmental conditions, and inadequate maintenance make electric motors prone to various failures. In this study, we propose a thermography-based motor fault detection method based on InceptionV3 model. To enhance the detection accuracy, we apply Contrast Limited Adaptive Histogram Equalization (CLAHE) to the input images. Furthermore, we improved the performance of the InceptionV3 by integrating a Squeeze-and-Excitation (SE) channel attention mechanism. The proposed model was tested using a dataset containing 369 thermal images of an electric motor with 11 types of faults. Image augmentation was employed to increase the data size and the evaluation was conducted using fivefold cross validation. Experimental results indicate that the proposed model can achieve accuracy, precision, recall, and F1 score of 98.82%, 98.93%, 98.82%, and 98.87%, respectively. Additionally, by freezing the fully connected layers of the InceptionV3 model for feature extraction and training a Support Vector Machines (SVM) to perform classification, it is able to achieve 100% detection rate across all four evaluation metrics. This research contributes to the field of industrial motor fault diagnosis. By incorporating deep learning techniques based on InceptionV3 and SE channel attention mechanism with a traditional classifier, the proposed method can accurately classify different motor faults.

## Introduction

Industrial motors play a crucial role in various manufacturing and production processes. However, they are susceptible to different types of faults that can lead to operational inefficiencies, production downtime, and increased maintenance costs. Therefore, accurate and timely fault diagnosis of industrial motors is of paramount importance for ensuring reliable and efficient operation^1^. Among the various motor fault detection methods, those relying on vibration analysis and thermal imaging have emerged as prevalent approaches. In a typical vibration-based condition monitoring system, multiple accelerometers are affixed to the machinery to capture vibration signals. These signals are subsequently processed to extract the informative features that enable precise fault localization ^2^. However, employing vibration analysis necessitates direct contact with the machine for sensor attachment. In contrast, thermal imaging offers a non-contact detection method, eliminating the need for physical contact with the motor and thereby avoiding potential hazards. This is particularly important for conducting motor health diagnosis during preventive maintenance^3^

In recent years, deep learning techniques have shown remarkable success in various computer vision tasks, including image classification^4–8^. Among these techniques, the InceptionV3 model has gained significant attention due to its ability to capture intricate spatial features through the use of inception modules^9^. Moreover, several studies have shown that attention mechanisms can be adopted in deep learning models to enhance feature representation by selectively attending to informative regions^10–12^. In particular, the Squeeze-and-Excitation (SE) channel attention mechanism has demonstrated its effectiveness in various vision tasks^13^.

We propose a novel approach that involves enhancing the original images using the Contrast Limited Adaptive Histogram Equalization (CLAHE)^14^ technique, followed by integrating the SE channel attention mechanism into the InceptionV3 model, and employing Support Vector Machine (SVM) classifiers to replace the classification layer in the model. Our approach aims to enhance the discriminative capability of the proposed method by employing CLAHE. This is achieved by preprocessing thermal images with CLAHE to enhance the visibility of features associated with faults. Additionally, we utilize the rich feature extraction capabilities of InceptionV3 and the enhanced attention mechanism provided by SE to improve the accuracy of fault classification in motor thermal images. Finally, by substituting the model's classification layer with an SVM classifier, we aim to enhance model representation and generalization, thus improving model performance.

To evaluate the performance of our approach, we conducted experiments using a publicly available dataset containing thermal images of an induction motor with 11 different types of motor faults, collected by the Electrical Machines Laboratory at the Babol Noshirvani University of Technology^15^. We trained the proposed model with the dataset to evaluated its classification performance. Furthermore, to explore the potential of incorporating traditional classifiers into our approach, we froze the fully connected layers of the InceptionV3 model and integrated a traditional classifier such as Support Vector Machines (SVM)^16^, K-Nearest Neighbors (KNN)^17^, and Random Forest (RF)^18^ to perform the classification. Experiments were conducted to evaluate the performance of these hybrid models for motor fault classification.

The contributions of this research include the development of a comprehensive fault classification approach for industrial motors that combines deep learning techniques with traditional classifiers. The proposed approach leverages the InceptionV3 model, SE channel attention mechanism, and CLAHE preprocessing to achieve accurate fault identification, which can have significant implications for effective industrial motors’ operation and maintenance.

The remainder of this paper is organized as follows: Sect. 2 provides a detailed description of the related work in fault classification and deep learning. Section 3 presents the methodology, including the architecture of the InceptionV3 model, the integration of the SE channel attention mechanism, and the CLAHE preprocessing technique. In Sect. 4, we present the experimental setup, test results and discussion. Finally, Sect. 5 summarizes the findings, and outlines future research directions.

## Related work

Electric motors consist of three main components: stator, rotor, and bearings. Failures in any of these components may lead to either mechanical or electrical faults. Mechanical issues such as bearing failures, rotor misalignment, bowed rotor, and rotor imbalance significantly impact motor performance, often stemming from excessive vibration, improper installation, or lubrication deficiencies. Electrical faults, resulting from thermal aging and unbalanced power supply, can lead to overheating and stator winding failures, posing considerable operational risks^19^. Usually, the health status of a motor can be diagnosed by examining changes in its conditions including temperature fluctuation^20,21^, vibration^22,23^, sound^24,25^ and current^26,27^. Among these methods, infrared thermography stands out as highly favorable due to its non-contact measurement capability.

Numerous methodologies have been proposed for the detection of motor faults using thermal imaging. For instance, Choudhary et al.^20^ developed a model to classify the condition of induction motors using thermal images. The model utilizes 2D-DWT, histogram analysis, PCA, Mahalanobis distance, and SVM for the classification. They showed that the model can accurately detect motors with inner or outer ring failure, and failure due to lack of lubrication. Almeida et al.^21^ employed a watershed transformation-based digital image processing algorithm to segment the thermal images of surge arresters into four categories. They then trained a neural fuzzy network using the thermal image data to classify the working states of the surge arrestors. They showed that the validation error of the system is approximately 10%.

Ramirez et al.^28^ proposed a method based on thermal image segmentation for motor fault detection. They manually segmented the thermal images into four layers and identified the hotspot regions and used them to determine the thermal characteristics of motors under different operating conditions. They performed motor fault detection by comparing the thermal coefficient indices. On the other hand, Huda et al.^29^ employed histogram-based statistics, gray level co-occurrence matrix, and component-based intensity to extract diverse features from the thermal images of electric equipment. These extracted features were then used to train a multilayer perceptron (MLP) network to classify different faults. Yang et al.^30^ proposed a thermal imaging-based rotating machinery fault diagnosis system. They enhanced the thermograms using bi-dimensional empirical mode decomposition (EMD) and extracted the histogram-based features. The feature dimension is then reduced using a generalized discriminant analysis and classification is performed based on a relevance vector machine. Their experimental results demonstrated the effectiveness of the approach in assisting rotating machinery fault diagnosis.

Eftekhari et al.^31^ proposed an algorithm for online non-destructive detection of stator winding inter-turn short circuit faults in asynchronous electric motors. Their proposed algorithm is based on extracting features from the infrared images indicating the hottest regions of the motor and analyzing these features to perform motor fault detection. Najafi et al.^15^ classification method to classify 11 different fault categories of motors. First, they applied a decision tree to divide the thermal images into two classes: hot and cold. Then, they used a random forest classifier to extract the regions of interest (ROIs) through a block-based approach. Finally, the fault classification task was accomplished using the random forest classifier. Lozanov et al.^32^ developed a framework that utilizes thermal images of induction motors to classify motor states into three categories: cooling fan fault, motor bearing fault, and healthy state. The framework employs Otsu thresholding to segment the moving structures and utilizes three texture features to represent the required information. The classification task is performed using an SVM classifier. and they showed that the approach can achieve 83.3% accuracy.

Besides traditional machine learning, some recent papers have also incorporated deep learning techniques for motor fault classification. For example, Khanjani and Ezoji^33^ utilized AlexNet for feature extraction, K-means algorithm for hot versus cold discrimination, and an SVM for classification. They achieved 100% accuracy in categorizing induction motor’s conditions. Ibrahim et al.^34^ used EfficientNetB0 and random forest for feature extraction and classification of thermal images, achieving 97% accuracy in categorizing different motor fault conditions. Sakalli and Koyuncu^35^ achieved 97.29% accuracy in classifying motor’s conditions using histogram-based features and random forest classifier. Furthermore, they showed that by utilizing the ResNet50 model to extract features, they are able to achieve 100% accuracy^36^.

Despite progress in fault detection and classification using thermal imaging, there are several challenges remain, including variations in environmental conditions, complex fault patterns, and the need for robust algorithms. In this study, we propose a novel approach that combines thermal imaging with deep learning techniques for accurate fault diagnosis of induction motors. By leveraging the advantages of deep learning algorithms in handling complex patterns and large datasets, we aim to improve the fault detection performance. CLAHE can alter the contrast of infrared images by computing local histograms and redistributing brightness^37^. It enhances the analysis of details, emphasizes features, and reduces noise, thus mitigating the impact of environmental variations on image quality.

## Proposed method

**Figure 1:**
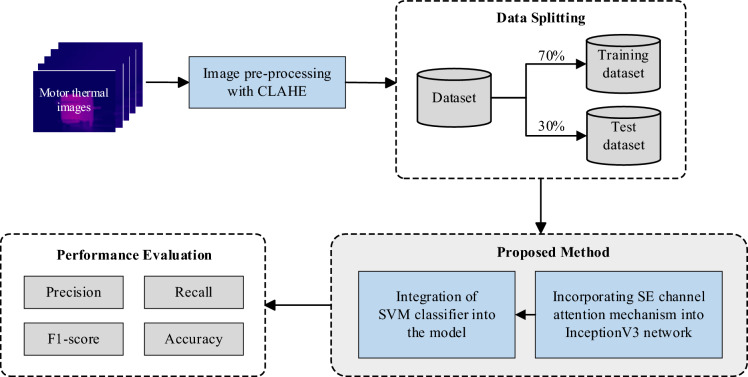
Experiment workflow diagram.

This study presents a new approach to motor fault detection, where InceptionV3 network is integrated with SE channel attention mechanism and SVM classifier to provide improved performance. This section begins by describing the dataset used in this study, followed by a brief introduction to the method of enhancing the data using CLAHE image preprocessing. Subsequently, the InceptionV3 model and SE block are explained, and finally, the process of integrating the SVM into the model is described. Figure 1 illustrates the methodology and workflow employed in this study.

### The dataset

**Figure 2:**
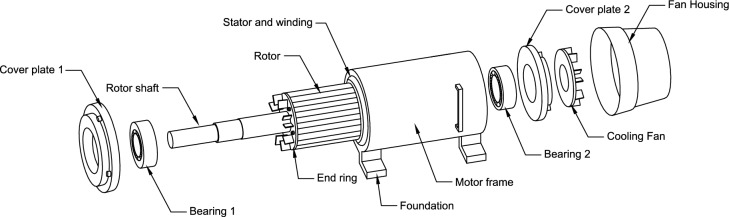
Structure of an induction motor.

This study used an industrial motor dataset created by Najafi et al. from the Electrical and Computer Engineering Laboratory at Babol Noshirvani University of Technology^15^. The dataset consists of 369 thermal images of three-phase induction motor under 11 types of fault conditions. The internal structure of the motor used in the creation of the dataset is illustrated in Fig. 2. Different fault conditions in the motor were studied. These include rotor blockage, cooling fan failure and various short-circuit conditions in the stator’s winding. The images were taken using a Dali-tech T4/T8 infrared thermal imager, and the image resolution is 320 × 240. Although high-resolution images are known to offer superior accuracy in fault detection, practical considerations must also be taken into account. Acquiring high-resolution thermal imaging equipment is often costly, and such images may capture irrelevant features, leading to time and cost inefficiencies. Therefore, one of our strategies is to enhance low-resolution images through technique such as image preprocessing and leveraging the model's feature extraction capabilities.

Table 1 shows the categories of motor faults and a sample image from each category in the dataset. The labels "A", "B" and "C" in the table represent the three phases of the induction motor, while SCT refers to the number of Short-Circuited Turns within the motor. The value of SCT is expressed as a percentage in this representation. The thermal images were captured from the motor operating under various conditions. These include short circuit in one phase with SCT of 10%, 30%, and 50%; short circuit in two phases with SCT of 10%, 30%, and 50%; short circuit in three phases with SCT of 10% and 30%; no-load; cooling fan failure; and rotor failure. It can be observed that as the number of faulty motor phases or the percentage of SCT increases, the thermal variation of the motor becomes more pronounced.

**Table 1:** The categories of motor’s faults in the dataset.

Category of fault	Description of fault	Percentage of the total image (number of images)	Number of images after augmentation	Sample images from the dataset
A&B50	Fault in phase A and B with 50% SCT	10.3% (38)	228	
A&B&C10	Fault in phase A, B and C with 10% SCT	8.4% (31)	186	
A&B&C30	Fault in phase A, B and C with 30% SCT	11.4% (42)	252	
A&C10	Fault in phase A and C with 10% SCT	8.4% (31)	186	
A&C30	Fault in phase A and C with 30% SCT	10.3% (38)	228	
A10	Fault in phase A with 10% Short-circuited Turns (SCT)	9.2% (34)	204	
A30	Fault in phase A with 30% SCT	10.0% (37)	222	
A50	Fault in phase A with 50% SCT	9.5% (35)	210	
Fan	Fault in motor’s fan	7.6% (28)	168	
Noload	Motor without load	6.8% (25)	150	
Rotor-0	Fault in rotor	8.1% (30)	180	

Based on the visual analysis, it is evident that the thermal variation in infrared images of motor under different fault conditions can provide crucial information for successful fault classification. However, it is also observed that for certain fault categories, there are significant similarities among the images. Additionally, the inclusion of rotor fault, fan fault, and no-load categories further complicates the differentiation of these fault types from the SCT faults. Therefore, the classification model must possess advanced feature extraction capabilities and a robust classification mechanism to effectively address this challenge. Considering these factors, we have selected the InceptionV3 model, not only because of its high accuracy in image classification but also because it has powerful feature extraction capabilities while keeping the model size relatively small. Additionally, we incorporated the SE channel attention mechanism into the model, enabling it to handle deeper features in the images more effectively and further enhancing the model's recognition accuracy. These qualities will contribute toward effective fault classification. The details of the proposed method will be elaborated in the subsequent sections.

### Image preprocessing using CLAHE

CLAHE is an extension of the traditional Histogram Equalization (HE) technique for image enhancement^38^. It is worth noting that the CLAHE technique offers several advantages for infrared thermography. It can enhance the details in infrared thermal images, making subtle temperature changes more visible. Additionally, it significantly increases the contrast of infrared thermal images, making the distinctions between different temperature regions more pronounced. Moreover, CLAHE can expand the dynamic range of infrared thermal images, resulting in a more uniform representation of temperature values in the image. These aspects contribute to better observation and analysis of heat distribution, as well as avoiding concentration of temperature information within a narrow range. This is particularly useful for detecting and analyzing detailed features such as heat gradients, hotspots, and temperature anomalies.

To apply CLAHE on an image, we adopt a channel-based approach. The image is decomposed into its color components using the Lab color space, where L represents the lightness channel, while a and b represent the chrominance channels. The steps involved in the CLAHE processing are as follows:

1. Convert the input image from RGB to Lab color space.
2. Separate the L, a, and b channels from the Lab image.
3. Apply CLAHE independently to the L channel using a specified block size and contrast limit.
4. Merge the enhanced L channel with the original a and b channels.
5. Convert the image back to RGB color space.

**Figure 3:**
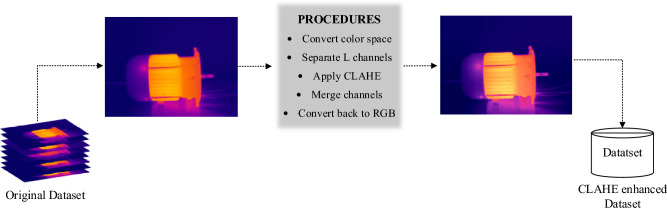
Process of applying CLAHE enhancement to the dataset.

**Figure 4:**
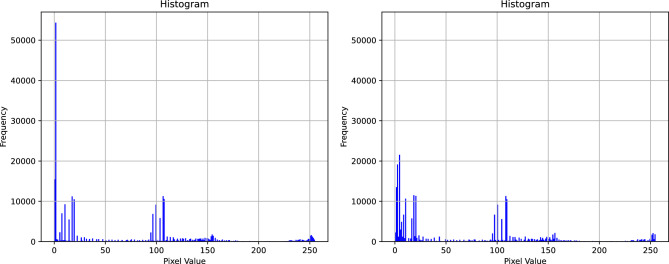
Histogram of the thermal image before and after applying CLAHE enhancement.

By performing CLAHE exclusively on the L channel, the image's lightness is selectively enhanced while preserving the original chrominance information. This approach ensures a balanced enhancement across the image, preserving the color representation. There are two important parameters to be set in the CLAHE algorithm: block size and contrast limit. We referenced the parameters used in the experiments by Farooq et al.^39^ and, considering our own experimental context, ultimately set the two parameters to 2 × 2 and 0.02 respectively. Figure 3 shows the process of applying CLAHE enhancement to the dataset. Figure 4 illustrates the histogram of the L channel for image labeled "A&B&C30" from Table 1, before and after applying the CLAHE enhancement.

### Architecture of the InceptionV3 model

The InceptionV3 model, also known as GoogLeNet^40^, is a widely used deep convolutional neural network architecture. It is renowned for its innovative inception module, which incorporates multiple convolutional filters of different sizes in parallel, allowing the network to capture both local and global features efficiently. This unique structure helps alleviate the vanishing gradient problem^41^ and enables effective feature extraction at various scales, which is the key advantage of InceptionV3. It utilizes multiple convolutional kernels of different sizes in parallel to process input data, enabling the capture of features across diverse scales. This enhances the network's ability to perceive objects and structures of different sizes. Additionally, InceptionV3 incorporates 1 × 1 convolutional layer in the initial module, which not only helps maintain a low parameter count but also provides powerful representational capabilities.

In the InceptionV3 network, the convolutional layer is one of its core components. The convolutional layer extracts feature from input data through convolutional operations and applies weighting and non-linear transformations to these features using convolutional filters and activation functions. The mathematical representation of the convolutional operation can be described using the convolutional operator formula:

1 $$\begin{array}{c}{y}_{i}={\sum }_{i}{x}_{i}*{w}_{ij}+{b}_{i}\end{array}$$

where ${x}_{i}$, ${w}_{ij}$, and ${b}_{i}$ represent the input, weights of the convolutional filter, and bias, respectively. In addition to the convolutional operation, the convolutional layer also employs an activation function to perform non-linear transformations on the output feature maps. ReLU (Rectified Linear Unit) is a commonly used activation function that helps alleviate gradient vanishing issues during training and improves the network's learning capability. The ReLU function is defined as:

2 $$\begin{array}{c}f\left(x\right)=\left\{\begin{array}{c}0,x<0\\ x,x>0\end{array}\right.\end{array}$$

**Figure 5:**
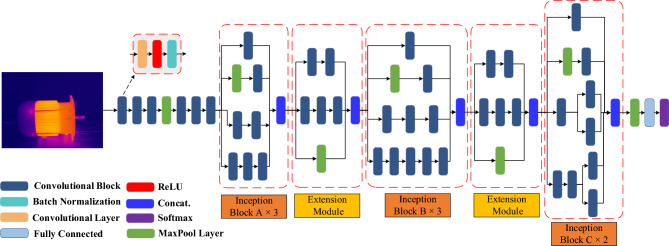
Structures of InceptionV3 network.

The unique architecture of InceptionV3 combines parallel convolutional filters, dimensionality reduction, and efficient resource management, which contributes to its effectiveness in handling various image datasets and addressing challenges related to computational resources. These features make InceptionV3 a popular choice in computer vision applications, particularly in tasks such as image recognition and object detection. The InceptionV3 network structure used in this paper is shown in Fig. 5.

**Figure 6:**
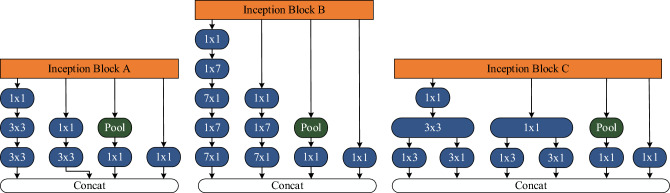
Three different initial modules in InceptionV3.

The inception block A, B and C are the initial modules of InceptionV3. These modules are constructed using 1 × 1 convolutional branch, 3 × 3 convolutional branch, 7 × 7 convolutional branch, and 1 × 1 convolutional branch with max pooling as shown in Fig. 6. The 1 × 1 convolutional branch is primarily used for dimensionality reduction and feature compression, contributing to a decrease in computational complexity and prevention of overfitting. The 3 × 3 convolutional branch is employed to capture features at a medium scale, aiding in detecting features such as shapes, edges, and textures. The 7 × 7 convolutional branch, on the other hand, is utilized to capture larger-scale features, facilitating the capturing of the overall structure and contextual information of the features.

#### Integration of SE channel attention mechanism

**Figure 7:**
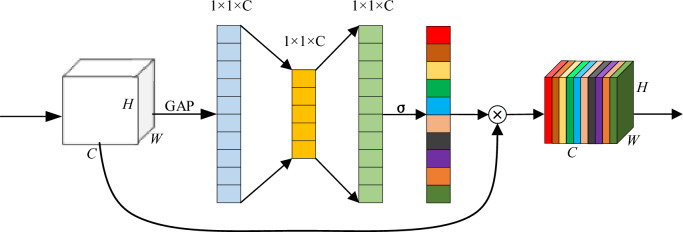
Structure of the SE block.

Squeeze-and-Excitation (SE) is a channel attention mechanism used to enhance the representational power of deep convolutional neural networks. It introduces a lightweight attention module in the network, which dynamically adjusts the importance of each channel in the input feature map through learning. Compression and excitation are the two key steps of the SE block, where these operations collectively enable the SE attention module to selectively amplify important features while suppressing less relevant ones. The structure of the SE block is shown in Fig. 7.

In the SE channel attention mechanism, several important equations are involved. Firstly, in the Squeeze operation, the input feature map of size *H* × *W* × *C* is compressed to 1 × 1 × *C* through global average pooling (GAP), where *H*, *W*, and *C* represent the height, width, and number of channels of the original feature map, respectively. This can be represented as follows:

3 $$\begin{array}{c}{z}_{c}=\frac{1}{H\times W}\sum_{i=1}^{H}\sum_{j=1}^{W}{u}_{c}\left(i,j\right)\end{array}$$

where ${z}_{c}$ represents the Squeeze output of the *c*-th channel, and *u*_*c*_ is the feature value of the *c*-th channel at position $(i,j)$ in the input feature map.

Secondly, in the Excitation stage, the relationships between channels and the generation of channel weights are learned through two fully connected layers and an activation function:

4 $$\begin{array}{c}s=\sigma \left({W}_{2}\delta \left({W}_{1}z\right)\right)\end{array}$$

where *s* represents the generated channel weight vector, ${W}_{1}$ and ${W}_{2}$ are learned weight parameters, *z* is the output vector from the Squeeze stage, $\delta$ denotes the ReLU activation function, and $\sigma$ represents the Sigmoid activation function.

Finally, by multiplying the channel weights with the original features, the feature representation after being processed by the SE attention mechanism is obtained:

5 $$\begin{array}{c}y={s}_{c}\cdot {u}_{c}\end{array}$$

Through experiments, it has been observed that the SE block can be placed at different positions within the InceptionV3 network, resulting in varying effects. In the proposed model, the SE block is placed after the Inception module to form the InceptionV3-SE structure. This will facilitate multi-scale feature fusion, enhance feature importance, and reduce computational complexity.

**Figure 8:**
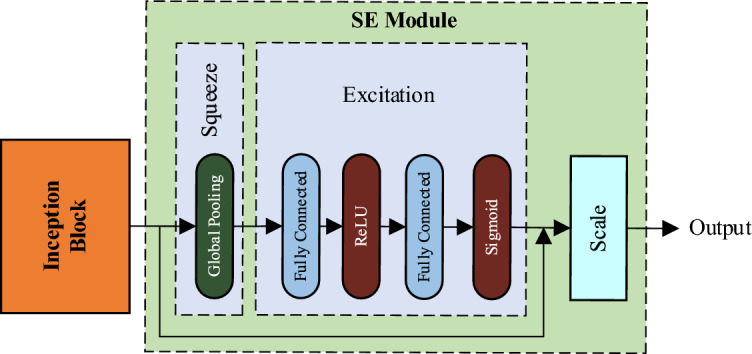
Structure of InceptionV3-SE.

In order to integrate the SE block with the InceptionV3, the dimension of the SE block’s input is modified to match the InceptionV3’s output. Similarly, the number of output channels of the SE block is adjusted to match the number of channels in the subsequent layer of the model. Figure 8 shows the integration of the Inception block and the SE module to form the proposed InceptionV3-SE model.

### SVM classification

SVM is a powerful supervised learning algorithm that can effectively handle classification problems ^42^. During the training phase, SVM searches for an optimal hyperplane to separate samples from different classes. The objective of SVM is to find a decision boundary that maximizes the margin between classes. Furthermore, we chose to use the One-vs-One SVM (OVO-SVM) approach to address the multi-class classification task. Specifically, we trained *N*(*N*-1)/2 binary classifiers, where *N* is the number of classes. Each binary classifier is used to distinguish between two different classes, and the final predicted class is determined by the number of victories.

We utilized a linear kernel function for the SVM. This will simplify the SVM’s computation of feature mapping, resulting in higher efficiency in training and prediction. The formula for the linear kernel function can be expressed as follows:

6 $$\begin{array}{c}K\left(x,y\right)={x}^{T}y\end{array}$$

where *x* and *y* represent the feature vectors of the input samples.

**Figure 9:**
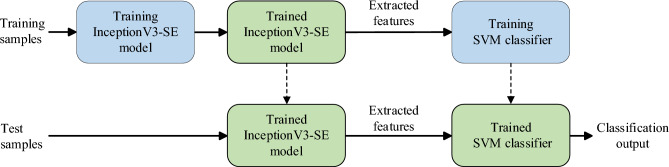
Structure of the proposed InceptionV3-SE-SVM model.

In this study, we investigated the effectiveness of using SVM as the main classifier instead of the classification layer in the InceptionV3. The architecture of the proposed InceptionV3-SE-SVM model was designed by replacing the fully connected and SoftMax layers of the InceptionV3-SE model with a linear SVM classifier. The high-level features extracted by InceptionV3-SE were utilized as inputs to the SVM classifier. By leveraging the rich features extracted by InceptionV3-SE, it will enhance the classification capability of SVM and improve the accuracy of motor faults classification. The structure of the InceptionV3-SE-SVM model is depicted in Fig. 9.

## Experiment result and discussion

### Experiment setup

**Figure 10:**
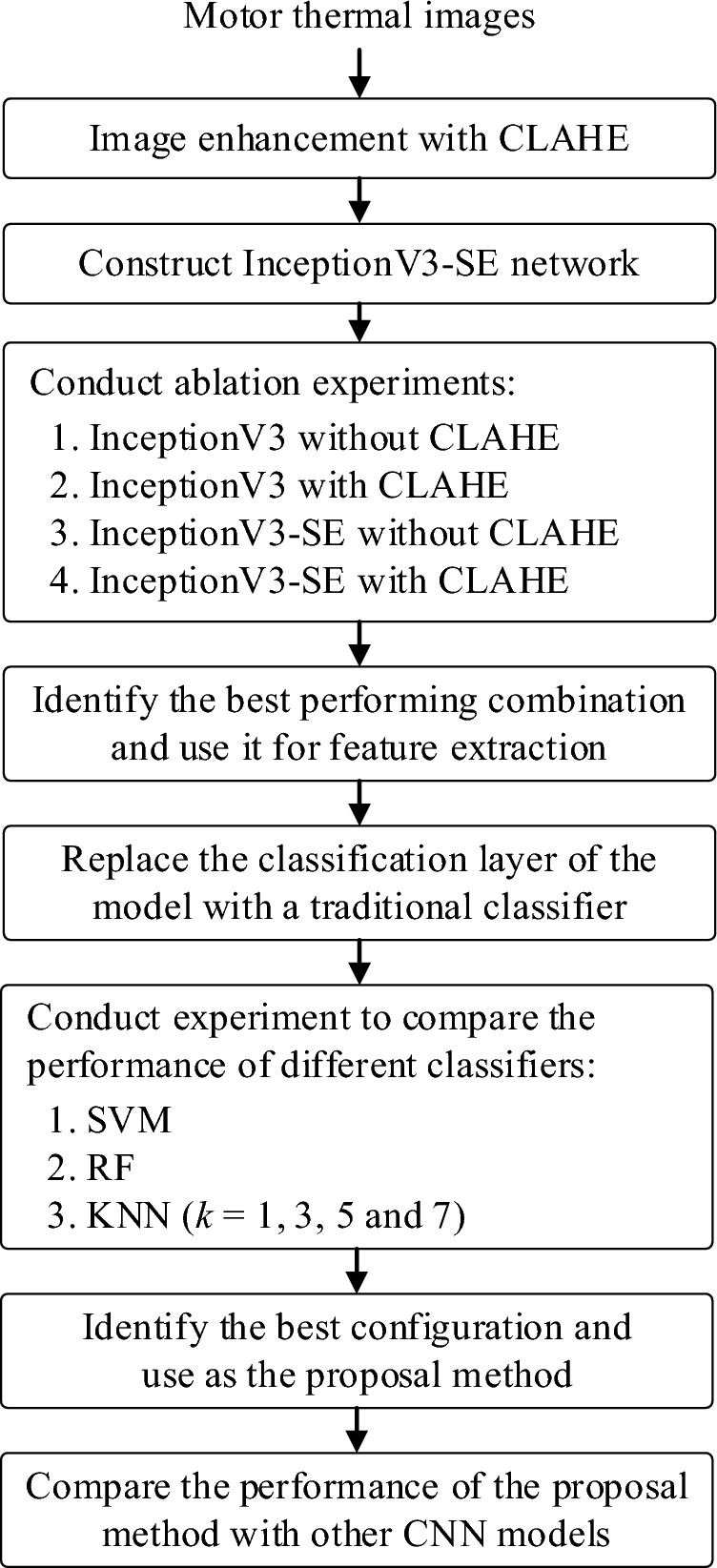
Implementation and experiments flowchart.

A series of experiments has been conducted to identify the best configuration for the proposed model and evaluate its performance. First, an ablation study was conducted to investigate the effectiveness of incorporating the SE channel attention mechanism and CLAHE in the InceptionV3 model. Next, experiments were conducted to study the performance of the model with the classification layer of InceptionV3 replaced with different traditional ML classifiers. Finally, we compared the performance of our proposed model with other CNN pre-trained models proposed by previous researchers. Figure 10 shows the flow of the implementation and the experiments conducted in this study.

This study utilized the Python programming language, specifically leveraging the PyTorch framework for deep learning tasks. All experiments were conducted using the PyCharm software on a personal computer equipped with a 2.9 GHz CPU, 16 GB RAM, and GeForce RTX 3060 graphics card.

Due to the limited size of the dataset, we apply data augmentation to increase the data size. This is done by performing image rotation, horizontal flipping, vertical flipping, and scaling. The augmented dataset comprised a total of 2214 images. Then, the augmented dataset was split into training and testing sets at a ratio of 70% to 30%. We employed fivefold cross-validation technique in our experiment to improve the reliability of the model’s performance evaluation and mitigate the risk of overfitting.

Table 2 presents the hyperparameter settings used in our model. We have conducted experiments to carefully tune these hyperparameters to strike a balance between model complexity and generalization performance, aiming to achieve the best possible results.

**Table 2:** Hyperparameter settings for the transfer learning models.

Parameter	Value/Range
Epoch	50
Batch size	32
Learning rate	0.00001
Optimizer	SGDM

### Performance metrics

In the experiments, we selected four metrics, namely Accuracy, Recall, Precision, and F1 score, to evaluate the performance of the models. These metrics provide quantitative measures to assess different aspects of the model's performance. The mathematical equations for these metrics are as follows.

7 $$\begin{array}{c}Accuracy=\frac{TP+TN}{TP+TN+FP+FN}\end{array}$$

8 $$\begin{array}{c}Recall=\frac{TP}{TP+FN}\end{array}$$

9 $$\begin{array}{c}Precision=\frac{TP}{TP+FP}\end{array}$$

10 $$\begin{array}{c}F1 Score=\frac{2\times \text{Precision}\times \text{Recall}}{\text{Precision}+\text{Recall}}\end{array}$$

In the equations, *TP, TN, FP* and *FN* represent the number of true positive, true negative, false positive and false negative predictions respectively. By evaluating the model's performance using these metrics, we can gain insights into its accuracy, its ability to correctly identify positive instances (recall), its ability to avoid false positive predictions (precision), and the overall balance between precision and recall (F1 score). These metrics provide a comprehensive assessment of the model's effectiveness in classifying different motor faults.

### Ablation studies on the proposed model

A series of experiments were conducted to identify the best configuration for the proposed model. These include evaluation on the model’s performance after incorporating the SE channel attention mechanism and using input images with and without CLAHE enhancement. We created and trained four models with different configurations: Original InceptionV3, InceptionV3 with SE block (InceptionV3-SE), InceptionV3 with CLAHE (InceptionV3-CLAHE) and InceptionV3 with SE block and CLAHE (InceptionV3-SE-CLAHE). The results are presented in Table 3.

**Table 3:** Results of ablation experiments.

	Module selection			Performance metrics			
Model	InceptionV3	SE block	CLAHE	Accuracy (%)	Precision (%)	Recall (%)	F1 Score (%)
InceptionV3	√	–	–	97.06	97.28	97.06	97.17
InceptionV3-CLAHE	√	–	√	97.87	98.13	97.87	98.00
InceptionV3-SE	√	√	–	98.69	98.79	98.69	98.74
InceptionV3-SE-CLAHE	√	√	√	98.82	98.93	98.82	98.87

From the results, it can be observed that applying CLAHE enhancement to the input images of the InceptionV3 model is beneficial for the classification task. However, the improvement achieved is limited to about 0.8%. In contrast, the InceptionV3 model incorporated with SE channel attention mechanism demonstrates outstanding performance in handling the given task. Compared to the baseline model consisting of the InceptionV3 model alone, the InceptionV3 model enhanced with the SE channel attention mechanism exhibits an average increase of 1.6% across all performance metrics. Nevertheless, using CLAHE enhanced images on the InceptionV3 with SE channel attention model gave a small improvement of approximately 0.2%. This shows that the InceptionV3 with SE block has possibly reached the upper limit of improvement achievable by CLAHE enhancement, due to inherent performance constraints. However, regardless of this limitation, the CLAHE enhancement still demonstrates a beneficial impact on the model’s performance. These findings suggest that while CLAHE enhancement provides a modest improvement in classification performance, the addition of the SE channel attention mechanism contributes a significant improvement to the overall performance of the InceptionV3 model.

**Figure 11:**
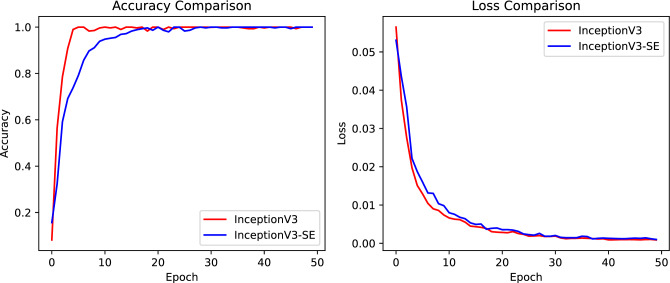
The validation accuracy and loss curves during the training of the InceptionV3 and InceptionV3-SE models.

The validation accuracy and loss curves during the training of InceptionV3 and InceptionV3-SE models are shown in Fig. 11. From the figure, it can be observed that InceptionV3-SE exhibits a faster learning speed compared to the InceptionV3 model. The accuracy curve of InceptionV3-SE is smoother and converges more rapidly than that of InceptionV3. The Loss curve for InceptionV3-SE also stabilized to a lower value more rapidly. This suggests that, in this task, the integration of the SE channel attention module to InceptionV3 will provide a better performance.

### Performance of the model using different classifiers

In the previous section, we identified that the InceptionV3 incorporated with SE block and using CLAHE enhanced images gives the best performance. In this section, we further improved the model by replacing the classification layer in the InceptionV3 with a traditional ML classifier. Experiments were conducted to evaluate the performance of using three different types of classifiers including SVM, KNN and Random Forest (RF). For the KNN classifier, the value of *k* represents the number of nearest neighboring data points selected, which determines the complexity and effectiveness of the classification model. Different values of *k* can lead to varying classification results and decision boundaries. In the experiment, the performance for four different values of *k* was evaluated. The results are shown in Table 4.

**Table 4:** Performance of the InceptionV3-SE-CLAHE model using different classifiers.

Types of classifiers		Accuracy (%)	Precision (%)	Recall (%)	F1 score (%)
KNN	*k* = 1	100.00	100.00	100.00	100.00
	*k* = 3	99.84	99.85	99.84	99.84
	*k* = 5	99.69	99.70	99.69	99.69
	*k* = 7	99.54	99.57	99.54	99.55
Random Forest		99.69	99.70	99.69	99.69
SVM		100.00	100.00	100.00	100.00

**Figure 12:**
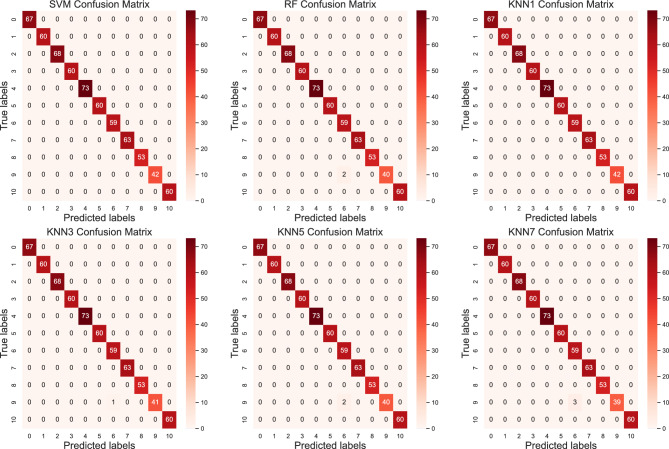
Confusion matrix for different classifiers.

From the results, it is evident that most of the machine learning classifiers performed well in this task, particularly the SVM classifier, which achieved 100% in all four performances metrics. The KNN classifier also demonstrated promising performance. When *k* equals 1, all four performances metrics of the model reached 100%. However, as *k* increased, the values of the metrics gradually decreased. We hypothesize that this may be due to the blurred boundaries between classes in our dataset and the imbalance in class distribution. This could make it more challenging for larger *k* values to capture local features between classes, resulting in a decrease in the model’s performance. On the other hand, the RF classifier exhibited moderate performance, with classification results similar to KNN with *k* = 5. Figure 12 shows the confusion matrix plots for different classifiers.

In the confusion matrix, the rows represent the actual categories, while the columns represent the model's predicted categories. There are 11 rows and columns, labelled from 0 to 10, which corresponds to the 11 fault types as listed in Table 1. The tests were conducted on the testing set, which consists of 33 fault images. From the figures, it can be observed that some classifiers incorrectly classify images of fault category 9 into fault category 6.

The findings from this experiment indicate that incorporating suitable traditional machine learning classifiers into the InceptionV3 model can be advantageous in facilitating accurate classification tasks. The SVM classifier, in particular, demonstrated exceptional performance across all evaluated metrics. The KNN classifier performed well in most cases, except for k = 5. The RF classifier showed relatively stable performance with marginal deviations from the original model. From the evaluation results, we proposed the best performing model based on InceptionV3 incorporated with SE block, CLAHE and SVM classifier (InceptionV3-SE-CLAHE-SVM) for the classification of motor faults.

In motor fault detection, time plays a crucial role. Rapid fault detection can prevent further damage to the motor and other related machinery, thereby minimizing downtime and expensive repairs. Therefore, an effective fault detection system not only needs to achieve high accuracy in fault detection but also needs to consider the time consumption. In our proposed method, comprising three key steps: CLAHE image enhancement, feature extraction using the InceptionV3-SE model, and classification employing SVM, we conducted tests on each step using 1000 images to assess the time required to complete each task. The test results are presented in Table 5.

**Table 5:** Time taken for each processing step in the proposed model.

Number of images	Time taken for each processing step			Total time (s)
	CLAHE (s)	Feature extraction (s)	SVM classification (s)	
1000	1.08	3.28	0.05	4.41

Table 5 illustrates that the average time required for processing 1000 motor thermal images is just 4.41 s, equivalent to 4.41ms per image. Such processing speed is sufficient for many real-time applications and capable of handling automated fault detection and real-time monitoring of electric motors.

### Comparison of the proposed model with other pre-trained deep learning models

**Figure 13:**
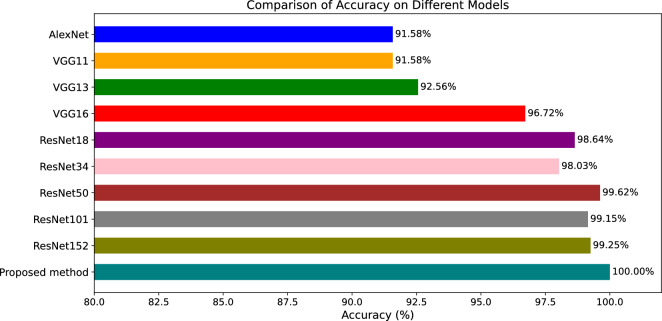
Performance comparison of the proposed method and other pre-trained deep learning models.

To demonstrate the superior classification performance of the proposed IncepsionV3-SE-CLAHE-SVM model, experiments were conducted to compare its performance with other prominent pre-trained deep learning models including AlexNet, VGG11, VGG13, VGG16, ResNet-18, ResNet-34, ResNet-50, ResNet-101, and ResNet-152. The results in terms of the classification accuracy are presented in Fig. 13.

From the graph, it can be observed that the proposed method achieved the best performance with accuracy of 100%. Among the pre-trained CNN models, ResNet-50 achieved the second highest accuracy of 99.62%. On the other hand, the accuracy of AlexNet and VGG11 models was relatively poorer, achieving only 91.58%. In general, the results show that the ResNet series models gives better performance compared to the VGG series and the AlexNet model.

Overall, the proposed method based on InceptionV3 incorporated with SE channel attention mechanism, SVM classifier and CLAHE image preprocessing delivers the best results in identifying different motor faults from thermal images.

## Conclusion

Industrial motor is a vital component in various industries. Precise and timely fault diagnosis of industrial motors is essential to mitigate the occurrence of unexpected failures and disruptions in operations. In this paper, an effective method for thermography-based motor fault detection is proposed. The method is based on the InceptionV3 model, with SE channel attention mechanism to enhance feature extraction and applying CLAHE to improve image’s contrast. Furthermore, a machine learning classifier is integrated into the model, taking the features extracted by the CNN as input to achieve higher classification accuracy. Evaluation results indicate that using CLAHE-enhanced images in the model leads to some performance improvement. On the other hand, employing the SE channel attention mechanism yields a significant improvement in the model’s performance. Additionally, it was found that, by using the SVM as the classifier, the performance of the model can be further improved. Experiment results show that the proposed method outperformed other pre-trained CNN models, achieving the best accuracy in the performance comparison. Hence, this method holds significant reference value in motor fault detection research for industrial applications. In the future, the proposed approach can be extended to multi-modal learning by incorporating acoustic and vibration signals, to improve the accuracy and robustness of motor fault detection.

## Data Availability

The dataset on thermal images of electric motor used in this study is available at https://data.mendeley.com/datasets/m4sbt8hbvk.
